# A scoring system to predict breast cancer mortality at 5 and 10 years

**DOI:** 10.1038/s41598-017-00536-7

**Published:** 2017-03-24

**Authors:** Esther Paredes-Aracil, Antonio Palazón-Bru, David Manuel Folgado-de la Rosa, José Ramón Ots-Gutiérrez, Antonio Fernando Compañ-Rosique, Vicente Francisco Gil-Guillén

**Affiliations:** 1 Surgery Service, General University Hospital of Elda, Elda, Alicante Spain; 2 Department of Clinical Medicine, Miguel Hernández University, San Juan de Alicante, Alicante Spain; 3 Surgery Service, Marina Baixa Hospital, La Vila Joiosa, Alicante Spain; 4 Department of Pathology and Surgery, Miguel Hernández University, San Juan de Alicante, Alicante Spain; 5 Research Unit, General University Hospital of Elda, Elda, Alicante Spain

## Abstract

Although predictive models exist for mortality in breast cancer (BC) (generally all cause-mortality), they are not applicable to all patients and their statistical methodology is not the most powerful to develop a predictive model. Consequently, we developed a predictive model specific for BC mortality at 5 and 10 years resolving the above issues. This cohort study included 287 patients diagnosed with BC in a Spanish region in 2003–2016. Main outcome variable: time-to-BC death. Secondary variables: age, personal history of breast surgery, personal history of any cancer/BC, premenopause, postmenopause, grade, estrogen receptor, progesterone receptor, c-erbB2, TNM stage, multicentricity/multifocality, diagnosis and treatment. A points system was constructed to predict BC mortality at 5 and 10 years. The model was internally validated by bootstrapping. The points system was integrated into a mobile application for Android. Mean follow-up was 8.6 ± 3.5 years and 55 patients died of BC. The points system included age, personal history of BC, grade, TNM stage and multicentricity. Validation was satisfactory, in both discrimination and calibration. In conclusion, we constructed and internally validated a scoring system for predicting BC mortality at 5 and 10 years. External validation studies are needed for its use in other geographical areas.

## Introduction

Breast cancer (BC) is the most frequently diagnosed tumor and the leading cause of cancer death in women in Spain, in Europe and in the world^1–4^. A multitude of prognostic factors provide information about the behavior of BC (demographic, clinical, pathological and molecular) and enable comparisons of case series and the results of different treatments. The most important of these factors are lymph node involvement, tumor size and grade^5–7^.

When assessing the prognosis of a disease such as BC, predictive models are very useful because they combine different risk factors to determine the likelihood of patient survival. Regarding predictive models of BC mortality, a meta-analysis published in 2006 found that of all those published, only the Nottingham Prognostic Index had sufficient scientific evidence for clinical use^7^. However, other predictive models assessing BC mortality were not included in this meta-analysis^8–12^. Table 1 shows the main features of the models and of the Nottingham Prognostic Index^8–15^. The majority of these models focused on all-cause mortality, and they were developed for specific populations (excluding advanced stages, considering specific treatments) or are difficult to use in clinical practice. Analyzing the methodology used for their development, we note that, generally, no survival models were used, risk groupings were made without justifying their classification and internal validation was not performed in the most correct way (Table 1)^8–15^. This validation should confirm two issues: discrimination and calibration. The first corresponds to the ability of the model to distinguish between patients who experience an event and those who do not, while the second assesses whether the probabilities of an event predicted by the model correspond to reality. There are different levels of calibration, with moderate being one of the most recommended (“a risk model is moderately calibrated if, among patients with the same predicted risk, the observed event rate equals the predicted risk”)^16^. We recommend validation through bootstrapping, since the other methods (split-sample and cross-validation) are less accurate^17^. To more accurately assess calibration, smooth curves are preferable (splines or loess transformations)^16, 18^. Not all models evaluated both discrimination and calibration, two of six models did not use bootstrapping for internal validation and none used smooth curves to assess calibration (Table 1)^8–15^. Consequently, we conducted a study to construct a predictive model specific to mortality at 5 and 10 years in patients diagnosed with BC without excluding any conditions, applying bootstrapping, and determining calibration through smooth curves (spline transformations). To facilitate use in routine clinical practice, the model was adapted to a points system and integrated into an application for the Android mobile phone operating system.

**Table 1:** Published predictive models for mortality in breast cancer patients.

Reference	Patients	*n*	Time (years)	Variables	Clinical issues	Methodological issues	Validation
Jimenez-Lee *et al*.^8^	BC stages 0–III	389	5	T, N and hormone receptors	Stage IV was excluded	The score for each variable was not justified and the definition of the risk groups was not justified	No
Chang *et al*.^9^	BC stages 0–III and follow-up >1 year	818	5	Age, diagnostic methods, tumor grade, N, hormone receptors and chemotherapy	Assessed all-cause mortality, stage IV was excluded, diagnostic methods are not useful, the model was not explained	They used a logistic regression model with censored data and the events-per-variable was less than 10.	AUC = 0.894; *p-value* (H-L) = 0.945
Fan *et al*.^10^	Invasive BC with mastectomy	1016	2 and 5	Age, T, N, M and estrogen receptors	Assessed all-cause mortality, a specific population and the model is laborious	No comment	C-statistic = 0.80; Calibration plot
Faneyte *et al*.^11^	BC without distant metastasis, <54 years, ≥4 positive lymph nodes, no previous other malignancies, treated with surgery and adjuvant therapy	739	2	Grade, estrogen receptors and N	Assessed all-cause mortality, a very specific population, the follow-up was very short and the model was not explained	They used a logistic regression model with censored data and it was not internally validated	No
Fontein *et al*.^12^	Postmenopausal, endocrine-sensitive and early BC	2602	5	Age, hormone receptors, grade, T, N, HER2, treatment and locoregional recurrence	Assessed all-cause mortality, a very specific population, early BC was not defined, the model is laborious to use and the web tool does not work*	Internal validation was not assessed with bootstrapping	C-index = 0.70–0.79; HSF = 0.995
The Nottingham Prognostic Index^13–15^.	Invasive primary operable BC with no other malignancies	387	5	T, N and grade	Assessed all-cause mortality and a specific population	The choice of cut-points was not explained, the risk groups changed in each publication and no bootstrapping	Graphically

AUC, area under the ROC curve; BC, breast cancer; HER2, human epidermal growth factor receptor 2; HSF, heuristic shrinkage factor; H-L, Hosmer-Lemeshow test. *Tested in October 2016.

## Methods

### Study population

Patients diagnosed with BC in the Elda Health Department were included. This Department provides health coverage to a region of southeastern Spain with a total of 184,195 inhabitants (2003). Health coverage is free and universal, for treatment of BC or any other disease.

### Study design and participants

This cohort study included patients diagnosed with BC in the Elda Health Department between 2003 and 2006 (inclusive). Patients were diagnosed through histological analysis by the Department of Pathology either through biopsy or surgical specimen. Patients were followed at the University General Hospital of Elda (the only hospital of this Health Department) from the date of diagnosis until July 2016 or the date of last contact with the patient, including death.

### Variables and measurements

The primary variable was BC-specific time-to-death. This variable was obtained in patient follow-up through the clinical history, which always includes the date of death and the cause, including BC. We must keep in mind that our variable considers each individual’s time to death from BC or no death from BC (last clinical contact or death from other causes), which is censored data^19^.

The secondary variables were collected at baseline (diagnosis date): age (years), personal history of breast surgery (none, benign pathology, or malignancy), personal history of any cancer, personal history of BC, premenopause, postmenopause, grade (low, intermediate/moderate or high)^20^, estrogen receptor, progesterone receptor, c-erbB2, TNM stage^21^, multicentricity/multifocality, and diagnosis (invasive ductal carcinoma, invasive lobular carcinoma, ductal/lobular *in situ*, or other). These study variables were selected because there is scientific evidence they are prognostic factors of BC^5, 7, 21–23^. Since a personal history of breast surgery affects the breast, it was suspected this could influence prognosis. Although not addressed as a prognostic factor in the scientific literature, it has been shown that patients with a personal history of BC who develop a second BC have a worse prognosis^24, 25^; thus, it is important to include this variable when developing a predictive model. All the secondary variables are easily obtained in clinical practice, which would facilitate their implementation in a predictive model.

Other factors to consider in the prognosis of breast cancer are the treatments given to the patient. For this reason the initial treatment was collected: neoadjuvant therapy, chemotherapy, hormone therapy, radiotherapy, breast surgery (none, mastectomy, or conserving) and lymphadenectomy. We note that sentinel lymph node biopsy does not appear in this list because it was introduced in our area after 2006.

### Sample size

Since the primary objective was to develop a predictive model, the sample size of our study had to verify that the events-per-variable was greater than 10^26^. Our final sample size was 287 women, of whom 55 died from BC, allowing construction of a predictive model with five predictors.

### Statistical methods

The qualitative variables were described by calculating absolute and relative frequencies and the quantitative variables through the mean and standard deviation. Given that our main variable was time-to-event, a multivariate Cox regression model was constructed with five predictors, verifying the assumption of proportional hazards by means of graphical and analytical tests^19^. Qualitative ordinal variables were considered quantitative predictors in the multivariate analysis (personal history of breast surgery, grade and TNM stage). Age was considered a quantitative continuous variable, as approximately 99% of our patient sample was over 35 years of age, which has been seen as a predictor of mortality^5^. These quantitative variables were analyzed linearly, as it was previously verified that they had no quadratic association with mortality (score test). Since we had a total of 22 possible predictors to introduce into the model, all combinations of 5, 4, 3, 2 and 1 predictors were determined, i.e., a total of 35,442. This approach is often called ‘Best-subsets’ model selection. In all of these, the optimism-adjusted estimate of the C-statistic was calculated and the combination yielding the highest result was chosen, i.e., the one with the highest discriminating capacity. Through the model estimated, the adjusted hazard ratios (HR) were obtained. The goodness of fit of this model was verified by the score test. The survival model was adapted to a points system using the Framingham Heart Study methodology^27^, which categorizes the predictors and, through a weighting system based on the model coefficients, gives a score to each category. The sum of the scores associated with each of the predictors gives a total score which correlates with a risk of event, so that the use in clinical practice of a points system is truly simple^27^. The points system methodology was used to calculate the risk of each total score at 5 and at 10 years from diagnosis of BC. To internally validate the points system we applied bootstrapping simulating 1000 samples as bootstrapping is the most recommended technique for this procedure^17, 28^ (although the relevant reference was published in 2001, its use is still recommended), and in each the C-statistic (discrimination) and the observed probability of event at 5 and 10 years was obtained (calibration) for each score through transformations by linear splines (smooth curves). These smooth curves are included in the recommended level of calibration (moderate)^16, 18^. A bootstrap sample is a random sample taken with replacement from the original sample with the same number of elements. The 1000 C-statistic values obtained were plotted on a histogram to analyze their distribution. We can say that the points system accurately discriminates the patient who died if the values of the distribution are high. The observed probabilities of mortality from BC obtained by smooth curves versus the probabilities predicted by the points system are represented on a Cartesian graph. To say that the model is well calibrated both probabilities should be very similar, i.e., the soft curve must conform to the diagonal line^16, 18^. Type I error was set at 5% for all analyses, and for each relevant parameter its associated confidence interval (CI) was calculated. All calculations were performed through IBM SPSS Statistics 19 and R 2.13.2.

### Ethical issues

The study was approved by the Ethics Committee of the Elda Department of Health. As this was a study of routine clinical practice without any intervention, informed consent was not requested from the patients. The Ethics Committee approved this procedure. The study was conducted in accordance with the basic principles of the Declaration of Helsinki World Medical Association and met the standards described in the guidelines for good clinical practice of the European Union.

### Mobile application

The points system was integrated into a mobile phone application using the Android operating system. Download of the application is free for all users from the store (*Google Play*) under the name *Breast Cancer Mortality*.

## Results

The study began with 292 patients, with five lost to follow-up, leaving a final sample size of 287 patients. With a mean follow-up of 8.6 ± 3.5 years, 55 women died of BC. This represents an incidence of 222 deaths per 10,000 person-years (95% CI: 167–289). The descriptive characteristics of the sample (Table 2) revealed the women had an average age of 59 years, 2.8% had a history of BC, and 65.9% were postmenopausal. Regarding the histological features, the majority were intermediate/moderate grade (40.8%), estrogen receptor-positive (74.6%), progesterone receptor-positive (63.1%), stage IV (4.9%) and with a diagnosis of invasive ductal carcinoma (78.4%). Treatments varied ranging from neoadjuvant therapy (6.3%) to surgery (89.5%) (Table 2).

**Table 2:** Descriptive characteristics and adjusted hazard ratios for predicting breast cancer mortality.

Variable	Total *n* = 287 n(%)/x ± s	Adjusted HR^†^ (95% CI)	*p-value*
Breast cancer mortality	55 (19.2)	N/A	N/A
Age	59.0 ± 14.6	1.02 (1.00–1.04)	0.119
Personal history of breast surgery:			
No	256 (89.2)	N/M*	N/M*
Benign pathology	22 (7.7)		
Malignancy	7 (2.4)		
Personal history of any cancer	19 (6.6)	N/M	N/M
Personal history of breast cancer	8 (2.8)	4.78 (1.82–12.57)	0.002
Premenopause	96 (33.4)	N/M	N/M
Postmenopause	189 (65.9)	N/M	N/M
Grade			
Low	65 (22.6)	2.26 (1.45–3.51)*	<0.001*
Intermediate/Moderate	117 (40.8)		
High	105 (36.6)		
Estrogen receptor	214 (74.6)	N/M	N/M
Progesterone receptor	181 (63.1)	N/M	N/M
c-erbB2	72 (25.1)	N/M	N/M
Stage			
0	13 (4.5)	1.83 (1.59–2.11)*	<0.001*
IA	93 (32.4)		
IIA	73 (25.4)		
IIB	45 (15.7)		
IIIA	27 (9.4)		
IIIC	3 (1.0)		
IV	14 (4.9)		
Neoadjuvant therapy	18 (6.3)	N/M	N/M
Chemotherapy	184 (64.1)	N/M	N/M
Hormone therapy	222 (77.4)	N/M	N/M
Radiotherapy	165 (57.5)	N/M	N/M
Multicentricity	50 (17.4)	1.60 (0.86–2.97)	0.140
Surgery			
No	30 (10.5)	N/M	N/M
Mastectomy	239 (83.3)		
Conserving	18 (6.3)		
Lymphadenectomy	257 (89.5)	N/M	N/M
Diagnosis			
Invasive ductal carcinoma	225 (78.4)	N/M	N/M
Invasive lobular carcinoma	38 (13.2)		
Ductal/lobular *in situ*	12 (4.2)		
Others	12 (4.2)		

CI, confidence interval; HR, hazard ratio; n(%), absolute frequency (relative frequency); N/A, not applicable; N/M, not in the multivariate model; x ± s, mean ± standard deviation. *Analyzed as a quantitative variable; ^†^the variables in the multivariate model are those with HR. Goodness-of-fit of the model: χ^2^ = 165.1, *p* < 0.001, C-statistic = 0.85 (standard error 0.039).

**Figure 1:**
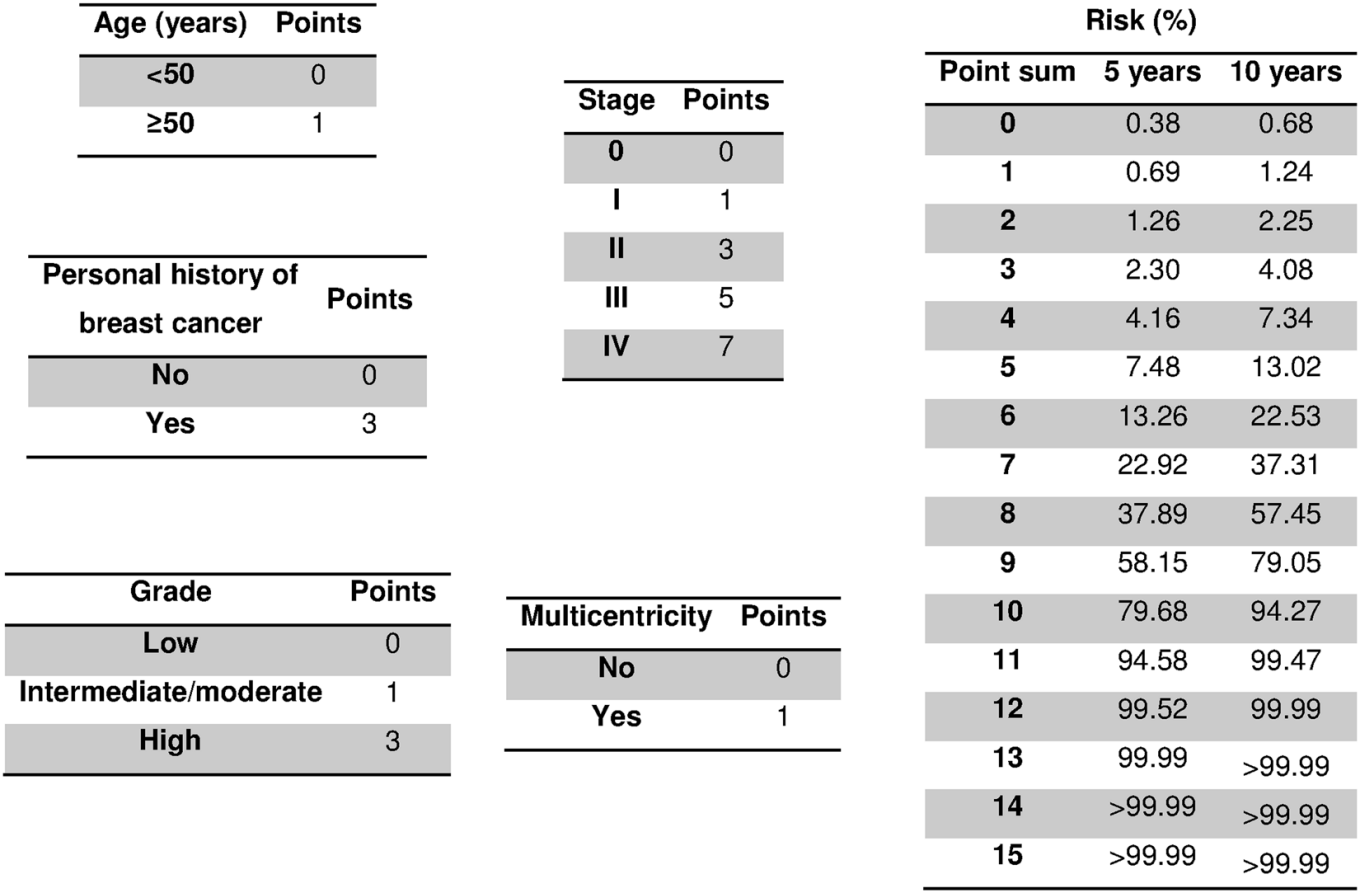
Scoring system to predict breast cancer mortality.

Analysis of 35,442 multivariate models provided a maximum C-statistic of 0.85 (standard error 0.039). Its associated model consisted of the following variables: older age (HR = 1.02, 95% CI: 1.00–1.04, *p* = 0.119), personal history of BC (HR = 4.78, 95% CI: 1.82–12.57, *p* = 0.002), higher grade (HR = 2.26, 95% CI: 1.45–3.51, *p* < 0.001), higher stage (HR = 1.83, 95% CI: 1.59–2.11, *p* < 0.001) and multicentricity (HR = 1.60, 95% CI: 0.86–2.97, *p* = 0.140). Adaptation to the points system model is shown in Fig. 1. As we can see, in the risk assessment there was apparently no difference between point sums 11–15. Nevertheless we have to take into account that differences did in fact exist, but they are given in decimals and after rounding the figures to 2 decimal places they appear to have the same risk. The reason they are similar is due to the exponentiality of the Cox model.

**Figure 2:**
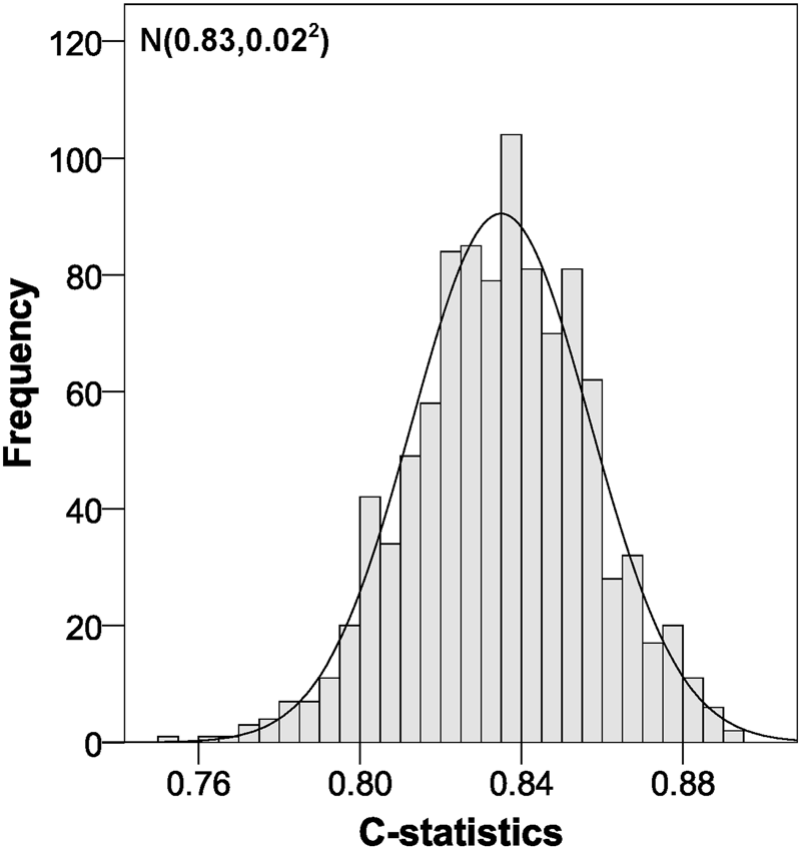
C-statistic distribution for the validation of our scoring system using the bootstrap method.

**Figure 3:**
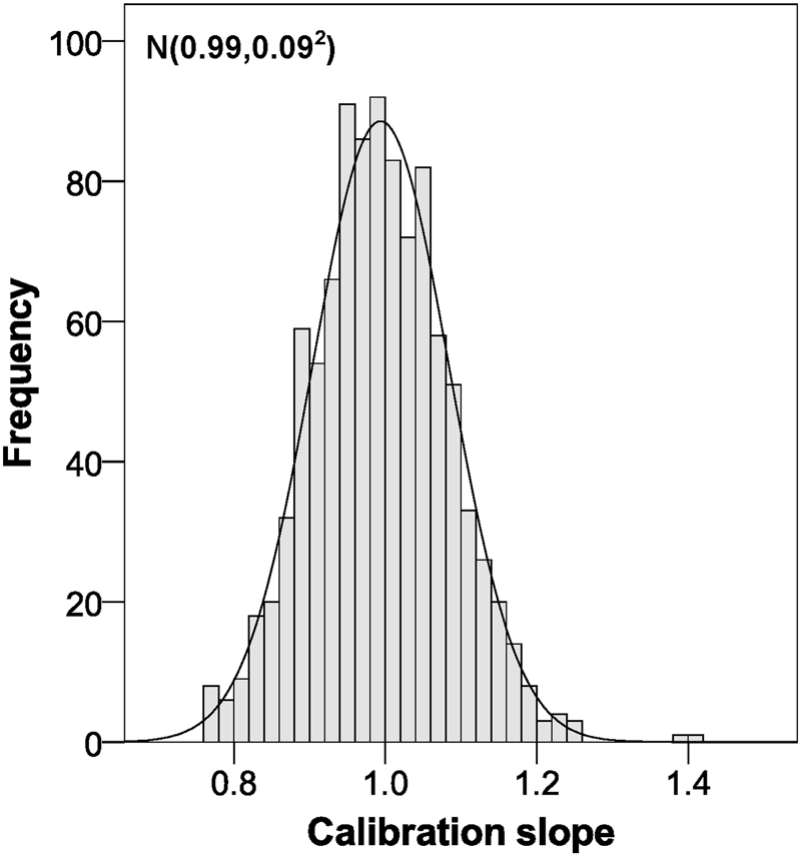
Calibration slope distribution for the validation of our scoring system using the bootstrap method.

**Figure 4:**
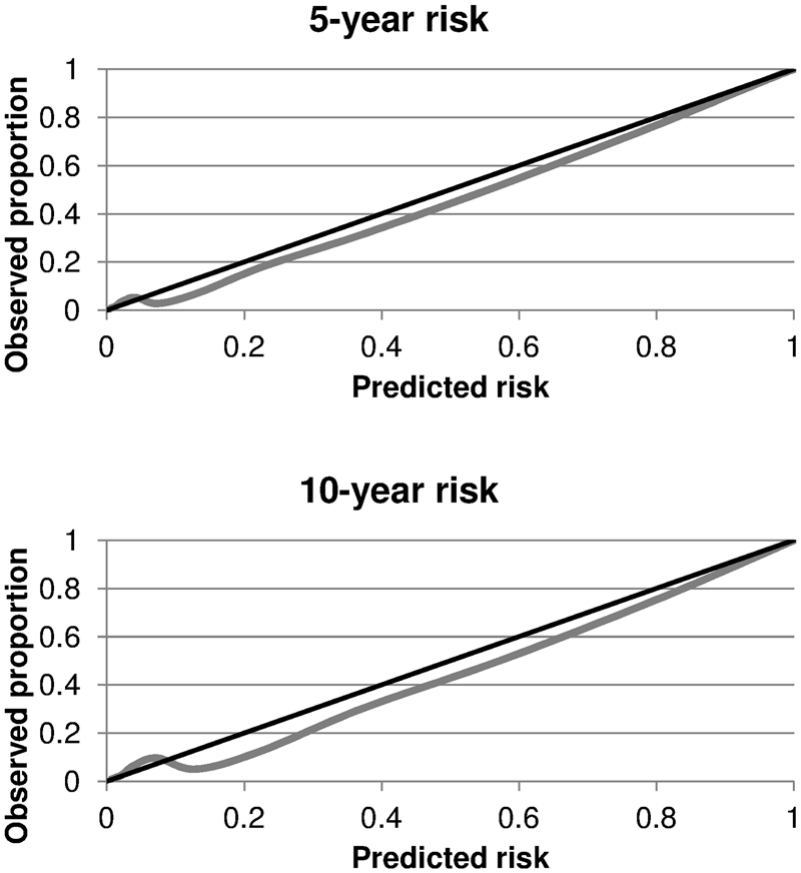
Smooth calibration plots for the validation of our scoring system using the bootstrap method. The black line represents perfect calibration and the grey line indicates the results of our calibration.

Internal validation through bootstrapping yielded a C-statistic of 0.83 (Fig. 2) and a slope within the linear calibration of 0.99 (Fig. 3). Smooth calibration curves at 5 and 10 years adjusted successfully to the diagonal line (Fig. 4). Although the observed risk may appear to be lower than the risk suggested by the model for most of the range of scores, it should be noted that in this type of calibration it is only necessary for the smooth curve to adjust to the straight line, whether it be oscillating, above or below^16^. In fact, the condition of having an underestimated risk exists in the calibration of survival models, even in very high sample sizes (1000 events), and the calibration is considered satisfactory when it does not deviate too far from the perfect condition^29^.

## Discussion

We constructed and internally validated a points system to predict mortality in BC at 5 and 10 years. The methodology followed used powerful statistical techniques for validating a predictive model: verification of more than 35,000 models, and discrimination considering censoring, bootstrapping and smooth calibration. The model can be applied to all patients with BC regardless of their clinical, histopathological or treatment characteristics, although it should be applied with caution in patients younger than 35 years, as our sample contained very few such patients.

The main strength of this study is the prediction model developed and internally validated (Table 1) that is applicable to all patients with BC (with caution in younger patients)^8–15^. This prediction model has improved upon existing models. Construction and internal validation were performed with the most accurate statistical techniques for predictive models. The predictor variables of our points system are easily obtained, allowing its routine use. Unlike other predictive models (Table 1)^8–15^, risk calculation using our model is very simple and is integrated in a mobile phone application that is free for all users of the Android operating system. This model fills the need for a predictive model for BC-specific mortality. Other models have generally been constructed to predict all-cause mortality.

Although it may seem that the sample size was insufficient to develop a predictive model, the size must be based on the events-per-variable ratio, which must be greater than 10^26^. This ratio was verified in our prediction model. Although the *p*-values associated with both age (*p* = 0.119) and multicentricity (*p* = 0.140) were not significant (*p* > 0.05), we must bear in mind that we did not assess each factor independently. All predictors were measured together, because our goal was to develop a prediction model. The model had high discriminatory capacity (C-statistic 0.85) and compared with the null model there were highly significant differences (χ^2^ = 165.1, *p* < 0.001). The model is therefore very satisfactory for prediction of mortality due to BC.

No other variables in the model evaluated in this study were prognostic factors (Table 2)^5, 7, 21–23^. Though other variables have been noted as prognostic factors they were not considered in this study as they were not collected correctly, such as the BRCA status. However, even without the inclusion of these other variables, our five predictors very successfully discriminated which patients would die of BC, in addition to successfully calibrating the model. Regarding selection bias, all patients with BC over a specific period were included in the study, i.e., none were excluded due to comorbidities, stage of disease or treatment received. Information bias was minimized through rigorous data collection. Additionally, the sentinel lymph node technique was not available when the baseline data for this study were recorded. Nonetheless, it should be recalled that this technique detects the presence of affected lymph nodes in the axilla and is therefore less aggressive than lymphadenectomy, though the resulting diagnosis is the same for both techniques. Consequently, this lack of inclusion of the sentinel lymph node among the study variables cannot be considered a limitation. Finally, concerning the duration of hormone therapy, it should be noted that our model is designed to predict mortality at the time of diagnosis. Thus, at this time we are unaware of the duration of hormone therapy, so we cannot include it as an explanatory variable in our scoring system. Whilst estrogen receptor-positive patients who receive hormone therapy have a better prognosis, we are estimating a multivariate model that considers this issue; that is, it correctly weights these factors in the patient prognosis. This can also be seen in other conditions, like cardiovascular diseases, where the general population does not have a very high prevalence of left ventricular hypertrophy (good prognosis), but the factor is still taken into account in the construction of the model, which can then be applied in the general population.

When we compare our model with those reported in the literature (Table 1)^8–15^, we note that they are generally applicable to selected populations, i.e., not all patients with BC in general. Additionally, some studies used models that did not consider censoring or the events-per-variable ratio^19, 26^, and constructed risk groups without any justification for their classification (Table 1)^8–15^. Regarding validation, some studies did not employ bootstrapping, when this is the most recommended technique^17^. In some papers discrimination was not analyzed and calibration, when it was done, was not done through smooth calibration (Table 1)^8–15^, which is the most accurate technique for this task^16^. When we combine all the features mentioned, no published model complies with all of them (Table 1)^8–15^: using a survival model, bootstrapping, analysis of events-per-variable, general population of patients with BC, discrimination considering censoring and calibration using smooth curves. Our model outperforms existing models in statistical methodology (development and validation). Finally, we stress that the outcome of our model is cancer-specific mortality, which is the most important consideration when establishing the prognosis of the disease.

The first factor in our points system was age, where risk of mortality increases with increasing patient age. This is consistent with the scientific literature, as our population contained few patients under 35 years^5^. The second factor was history of BC, which increases the risk of mortality. As previously mentioned (*Variables and measurements*), a second BC has been shown to worsen the prognosis^24, 25^. As expected, the stage and grade were associated with higher mortality, consistent with other predictive models and a large number of studies (Table 1)^5, 8–15, 20, 21^. Finally, we found that multicentricity indicated a greater likelihood of death. This is consistent with other studies, which found that this was an independent prognostic factor^30^.

Our points system can be applied quickly in routine clinical practice to any patient with BC using simple parameters (using the points system integrated into the mobile application). With this we can easily determine the prognosis and inform the patient. We encourage other authors to validate our points system in other populations, and to employ the methodology followed here to develop predictive models in patients with BC, as well as with other types of cancer or other diseases. Although our model adjusts our data well, it could nevertheless be over-estimating; accordingly, its calibration and discrimination need to be confirmed with other datasets in other areas, and which include patients of different races and ethnicities. Finally, another interesting line of research concerns comparing the prognostic power of our model with that of other models already published (Table 1). This, though, would require cohorts with large sample sizes including at least 100 events and 100 non-events^16^.

## Conclusion

A points system for predicting mortality due to BC at 5 and 10 years has been constructed and internally validated. A mobile application in Android has been designed and is available for use. To use our forecasting system in other populations, external validation studies are needed.
